# Penicillin and Cefotaxime Resistance of Quinolone-Resistant *Neisseria meningitidis* Clonal Complex 4821, Shanghai, China, 1965–2020

**DOI:** 10.3201/eid2902.221066

**Published:** 2023-02

**Authors:** Mingliang Chen, Youxing Shao, Jiayuan Luo, Lingyue Yuan, Minggui Wang, Min Chen, Qinglan Guo

**Affiliations:** Shanghai Institutes of Preventive Medicine, Shanghai, China (Mingliang Chen);; Shanghai Municipal Center for Disease Control and Prevention, Shanghai (Mingliang Chen, J. Luo, L. Yuan, Min Chen);; Huashan Hospital, Fudan University, Shanghai (Y. Shao, M. Wang, Q. Guo);; Key Laboratory of Clinical Pharmacology of Antibiotics, National Heath Commission of the People's Republic of China, Shanghai (Y.Shao, M.G. Wang, Q. Guo)

**Keywords:** Neisseria meningitidis, penicillin, third-generation cephalosporins, antimicrobial resistance, clonal complex 4821, CC4821, horizontal gene transfer, multidrug resistance, bacteria, Shanghai, China

## Abstract

Clonal complex 4821 (CC4821) *Neisseria meningitidis*, usually resistant to quinolones but susceptible to penicillin and third-generation cephalosporins, is increasing worldwide. To characterize the penicillin-nonsusceptible (Pen^NS^) meningococci, we analyzed 491 meningococci and 724 commensal *Neisseria* isolates in Shanghai, China, during 1965–2020. The Pen^NS^ proportion increased from 0.3% in 1965–1985 to 7.0% in 2005–2014 and to 33.3% in 2015–2020. Of the 26 Pen^NS^ meningococci, 11 (42.3%) belonged to the CC4821 cluster; all possessed mutations in penicillin-binding protein 2, mostly from commensal *Neisseria*. Genetic analyses and transformation identified potential donors of 6 *penA* alleles. Three Pen^NS^ meningococci were resistant to cefotaxime, 2 within the CC4821 cluster. With 96% of the Pen^NS^ meningococci beyond the coverage of scheduled vaccination and the cefotaxime-resistant isolates all from toddlers, quinolone-resistant CC4821 has acquired penicillin and cefotaxime resistance closely related to the internationally disseminated ceftriaxone-resistant gonococcal FC428 clone, posing a greater threat especially to young children.

*Neisseria meningitidis* colonizes the pharynx of humans and is responsible for severe invasive meningococcal diseases (IMD), such as septicemia and meningitis; case-fatality rate for IMD is ≈11% (*1*). *N. meningitidis* can be divided into 12 serogroups, and evolutionary relationships among isolates from within and without serogroup can be described by clonal complex (CC), defined by multilocus sequence typing (MLST) (*2*). The distribution of serogroups and CCs varies by time and geographic location.

In the past 20 years in China, *N. meningitidis* serogroup C (NmC) CC4821 has replaced *N. meningitidis* serogroup A (NmA) CC5 as being predominant nationwide (*3*–*6*). This replacement was driven by national dissemination of a hyperinvasive and quinolone-resistant clone within CC4821, China^CC4821-R1-C/B^, and led to the high frequency of resistance (≈70%) of meningococci in China against fluoroquinolones, which had been used as antimicrobial prophylaxis for close contacts of IMD patients since 2005 (*5*).

CC4821 is expanding worldwide and has been found in 19 countries outside of China (*7*); infections include urogenital and anorectal infections among men who have sex with men in Europe (*8*). Global CC4821 diverges into 4 sublineages, of which a high proportion (79.3%) of CC4821 isolates in Europe and in North and South America possess molecular markers of nonsusceptibility to penicillin (Pen^NS^). In contrast, the proportion was much lower in China (10.5%) (*7*).

In several countries, the first-line therapeutic antimicrobial therapies for IMD have been penicillin and third-generation cephalosporins (3GCs), such as cefotaxime and ceftriaxone (*9*); long-term meningococcal chemoprophylaxis for patients using complement inhibitors includes penicillin (*10*). Because IMD can cause death within hours (*11*), the frequency of infections with *N. meningitidis* resistant to penicillin and 3GCs is an issue of great concern worldwide.

*N. meningitidis* resistance to 3GCs is rare, and only 1 cefotaxime-resistant isolate has been reported in the United Kingdom (*12*). In recent years, Pen^NS^ meningococci have become more frequent worldwide (*13*,*14*), but data for meningococci from China with Pen^NS^ and 3GCs resistance remain poorly described. Two studies from the China Center for Disease Control and Prevention (China CDC) showed that the Pen^NS^ proportion was 4.9% during 2003–2012 and 15.2% during 2005–2019 nationwide and that 2.6% of isolates showed intermediate resistance to cefotaxime (without MIC values) during 2005–2019 (*15*,*16*). A provincial study from Zhejiang showed a Pen^NS^ proportion of 51.4% during 2011–2021 (*17*). However, information regarding the resistance mechanism and the genetic origin is unavailable. On the basis of *N. meningitidis* and commensal *Neisseria* isolates in Shanghai, China, since 1965, our aim with this study was to report the proportion and clonal relationship of Pen^NS^ isolates, demonstrate the origin and evolutionary changes of their *penA* genes, and evaluate the role of CC4821 in disseminating penicillin and 3GC resistance.

## Materials and Methods

### Isolate Collection

During 1965–2020, a total of 491 meningococcal and 724 commensal *Neisseria* isolates were collected in Shanghai. The meningococci were isolated from 171 IMD patients and 320 asymptomatic carriers during 1965–1985 and 2005–2020 (*5*), and the commensal *Neisseria* isolates were isolated from healthy persons during 2013 and 2019 (*18*).

### Antimicrobial Susceptibility Testing

Using the agar-dilution method, we determined MICs of penicillin, azithromycin, cefotaxime, ceftriaxone, meropenem, chloramphenicol, ciprofloxacin, minocycline, rifampin, and trimethoprim/sulfamethoxazole. Using antibiotic gradient strip diffusion methods (Etest; bioMérieux, https://www.biomerieux.com), we determined the MICs for the Pen^NS^ isolates and cefotaxime-resistant isolates. We interpreted breakpoints according to the 2022 guidelines of the Clinical and Laboratory Standards Institute (*19*).

### *N. meningitidis* Isolate Typing

We determined the serogroup of *N. meningitidis* isolates by using slide agglutination with monoclonal antiserum (Remel Europe Ltd., www.remel.com). All isolates were analyzed by MLST and typing for PorA and FetA according to previously described protocols (*3*). We analyzed whole-genome sequences of the Pen^NS^ meningococci by using the meningococcal core-genome MLST (cgMLST) schemes of *N. meningitidis* cgMLST version 1.0 for Pen^NS^ meningococci and the L44 cgMLST schemes for CC4821 isolates (*7*,*20*).

### Analysis of Penicillin and 3GC Resistance–Associated Genes

Low-level penicillin resistance and 3GC resistance of *N. meningitidis* are mainly associated with mutations in the penicillin-binding protein (PBP) 2, which can be determined by sequencing its coding gene, *penA*, using the primers recommended by Taha et al. (*14*). On the basis of a 402-bp fragment (nucleotides 1321–1722) encoding transpeptidase domain (*14*), we determined the *penA* alleles according to the nomenclature in the *Neisseria* PubMLST database (*21*). We submitted novel *penA* alleles discovered in this study, and they were assigned new allele numbers in the database. The *ponA* gene encoding PBP1, in which the mutation L421P was reportedly associated with penicillin resistance in *N. gonorrhoeae*, was analyzed as previously described (*22*). We performed phylogenetic analyses via maximum-likelihood analysis with IQ-TREE version 2.2.0 (*23*), using the 402-bp *penA* sequences collected in this study and those in the *Neisseria* PubMLST database from different *Neisseria* species and different countries, deposited before December 25, 2021 (*21*).

### Determination of Potential Donors and Recombination Crossover Points of Meningococcal *penA* Alleles

On the basis of previously described criteria, we considered a commensal *Neisseria* strain to be a potential donor for a recombinant *penA* allele in *N. meningitidis* (*18*). To identify the donors and the crossover points, we performed Illumina sequencing (https://www.illumina.com) on representative *N. meningitidis* and commensal *Neisseria* isolates that shared a candidate recombinant *penA* allele. We checked combination crossover points identified by visual inspection by using RDP software (Recombination Detection Program), version 4.97 (*24*).

### Genetic Transformation

We performed the transformation of chromosomal DNA (500 ng) and *penA* fragment (100 ng) from *Neisseria* donor isolates into *N. meningitidis* as previously described (*18*). We selected 3 transformants of each pair of donor and recipient isolates for further study. We determined the penicillin MICs by using Etest and the genomes of the transformants by using Illumina sequencing. We submitted the genomes of *N. meningitidis* and commensal *Neisseria* that were sequenced in this study to PubMLST *Neisseria* Database with PubMLST identification numbers (Appendix 1 Table 1).

## Results

### Increased Penicillin Nonsusceptibility of Meningococcal Isolates 

**Figure 1:**
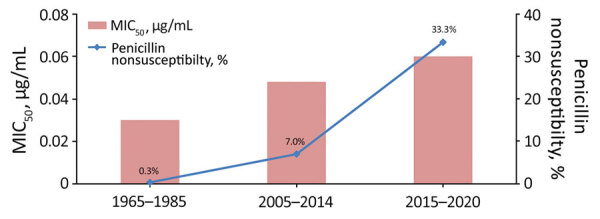
Percentage of meningococcal isolates with penicillin nonsusceptibility and MIC_50_ values, Shanghai, China, 1965–2020. MIC_50_, minimum inhibitory concentrations at which 50% of the tested isolates are inhibited.

A total of 491 isolates were available from IMD patients and asymptomatic carriers in Shanghai during 1965–2020. The predominant serogroup of isolates causing IMD shifted from *N. meningitidis* serogroup A (NmA) (72.6%, 90/124) in 1965–1985 to *N. meningitidis* serogroup C (NmC) 42.6%, 20/47) and *N. meningitidis* serogroup B (NmB) (40.4%, 19/47) in 2005–2020. NmB sustained prevalence in carriage isolates in both periods, and NmA and NmC decreased markedly more in carriage isolates during 2005–2020 than 1965–1985 (Appendix 2 Figure 1).

Antimicrobial susceptibility tests showed that 26 (5.3%) isolates were Pen^NS^, of which 3 isolates (Nm462, Nm463, and Nm507) were also resistant to cefotaxime (MIC range 0.25–0.50 μg/mL). The average proportion of penicillin nonsusceptibility was 3.5% (6/171) for IMD isolates and 6.3% (20/320) for carriage isolates, showing a total increase from 0.3% (1/303) in 1965–1985 to 7.0% (10/143) in 2005–2014 and to 33.3% (15/45) in 2015–2020 (Table). Correspondingly, the MIC at which 50% of tested isolates are inhibited (MIC_50_) for the 3 periods increased from 0.03 μg/mL in 1965–1985 to 0.048 μg/mL in 2005–2014 and to 0.06 μg/mL in 2015–2020 (Figure 1). The average proportions of nonsusceptibility during 1965–2020 were 26.3% (129/491) for ciprofloxacin and 93.9% (461/491) for trimethoprim/sulfamethoxazole. All isolates were susceptible to azithromycin, ceftriaxone, meropenem, chloramphenicol, minocycline, and rifampin.

**Table:** Frequency of identification of penicillin-nonsusceptible meningococcal isolates, Shanghai, China, 1965–2020*

Isolate source	Total	1965–1985	2005–2020		
Total	2005–2014	2015–2020
Total	26/491 (5.3)	1/303 (0.3)	25/188 (13.3)	10/143 (7.0)	15/45 (33.3)
IMD, n = 171					
Average	6/17 (3.5)	0/124 (0)	6/47 (12.8)	1/38 (2.6)	5/9 (55.6)
Age <18 y	4/81 (4.9)	0/46 (0)	4/35 (11.4)	1/29 (3.4)	3/6 (50)
Age >18 y	2/90 (2.2)	0/78 (0)	2/12 (16.7)	0/9 (0)	2/3 (66.7)
Carriage, n = 320					
Average	20/320 (6.3)	1/179 (0.6)	19/141 (13.5)	9/105 (8.6)	10/36 (27.8)
Age <18 y	12/51 (23.5)	0/8 (0)	12/43 (27.9)	3/22 (13.6)	9/21 (42.9)
Age >18 y	8/269 (3.0)	1/171 (0.6)	7/98 (7.1)	6/83 (7.2)	1/15 (6.7)

*Values are no./no. nonsusceptible isolates (%). IMD, invasive meningococcal disease.

### Epidemiologic and Molecular Characterizations of Pen^NS^ Isolates

Of the Pen^NS^ isolates, NmB was predominant (69.2%, 18/26; Appendix 1 Table 2). Except for 14 isolates unable to be assigned to any CCs (singletons), 8 isolates were assigned to CC4821 and another 4 each were assigned to a different CC. Nineteen (73.1%) Pen^NS^ isolates also showed quinolone resistance (MIC range 0.06–0.5 μg/mL), representing 13 *gyrA* alleles harboring GyrA mutations (T91I, n = 16; D95N, n = 3).

**Figure 2:**
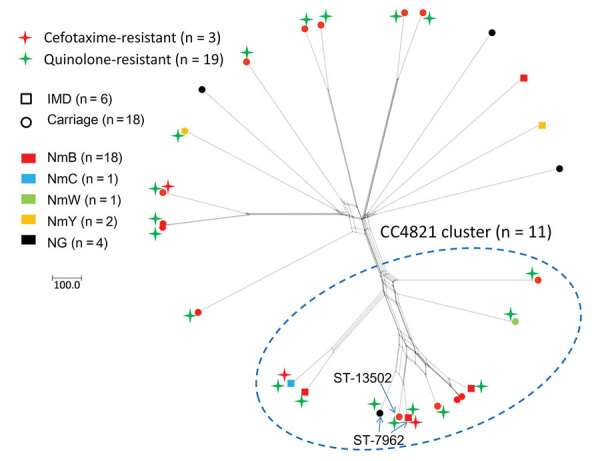
Allele-based clusters of penicillin-nonsusceptible meningococcal isolates identified by using *Neisseria meningitidis* core-genome multilocus sequence typing (MLST) v1.0 scheme, Shanghai, China, 1965–2020. Arrows indicated the 3 singleton isolates identified by 7-locus–based MLST but assigned to CC4821 cluster by core-genome MLST analysis. Scale bar indicates numbers of loci. CC, clonal complex; IMD, invasive meningococcal disease; NG, nongroupable; NmB, serogroup B; NmC, serogroup C; NmW, serogroup W; NmY, serogroup Y; ST, sequence type.

Genome analysis showed that 11 Pen^NS^ isolates were clustered together, of which 8 isolates belonged to CC4821 and 3 were singletons (2 ST-7962 and 1 ST-13502, each shared 4 loci with a CC4821 ST-5664) (Figure 2), so the cluster was designated as CC4821 cluster. We located the 11 Pen^NS^ CC4821 cluster isolates within the known 4 global CC4821 sublineages (*7*) and found that the L44.2 sublineage was predominant (n = 7), followed by L44.1 (n = 2), L44.3 (n = 1), and L44.4 (n = 1) (Appendix 2 Figure 2).

### Characteristics of Cefotaxime-Resistant Isolates

The 3 Pen^NS^ and cefotaxime-resistant isolates, recovered during 2017 and 2019, displayed reduced susceptibility to ceftriaxone (0.064–0.125 μg/mL) compared with the wild-type strain NM040 (<0.002 μg/mL). They all conferred resistance to ciprofloxacin, harboring the T91I mutation in GyrA. Two invasive isolates were assigned to the CC4821 cluster (L44.1 and L44.2; Figure 2) with different characterizations (Appendix 2 Table 1). Harboring different *penA* alleles (*penA777*, *penA795*, and *penA865*), they all possessed 5 penicillin-resistance-associated mutations (F504L, A510V, I515V, H541N, and I566V) and mutations associated with reduced cephalosporin susceptibility, including A311V, I312M, V316T, T483S, and G545S in the C-terminal or penicillin-binding domain of PBP2.

### Evolution of Meningococcal *penA* Alleles 

The *penA* alleles were obtained from all 491 *N. meningitidis* isolates. From the 465 penicillin-susceptible isolates, we identified 22 *penA* alleles; the most frequently isolated alleles were *penA1* (27.5%, 135/491), *penA4* (23.8%, 117/491), *penA3* (14.5%, 71/491), and *penA83* (10.2%, 50/491). Most of those isolates (95.7%, 445/465) harbored the *penA1* allele or an allele with a deduced amino acid sequence identical to the *penA*1 allele. The allele *penA83* was prevalent only during 1973–1985 and was almost always possessed by the NmA CC1 epidemic clone of that period (94%, 47/50). In contrast, both *penA4* and *penA1* were prevalent in the 3 periods; percentages were 52.6% (159/302) during 1965–1985, 59.4% (79/133) during 2005–2014, and 46.7% (14/30) during 2015–2020.

In the 26 Pen^NS^ isolates, we found 20 *penA* alleles, in which 18 alleles possessed the 5 common PBP2 mutations, 1 allele (*penA866*) possessed only 2 mutations (F504L and A510V), and another allele (*penA184*) harbored none of the 5 mutations but had an A549T mutation in the transpeptidase region (amino acid sites 441–574). Except for the alleles *penA405* (n = 3 isolates), *penA293* (n = 2), *penA552* (n = 2), *penA832* (n = 2), and *penA843* (n = 2), another 15 alleles were each possessed by only 1 isolate. Allele *penA405* was carried by 3 penicillin-intermediate CC4821 isolates of L44.2, and the other alleles were scattered in various CCs or singletons (Figure 2; Appendix 2 Figure 2). No isolates possessed mutations in the *ponA* gene of nucleotides 1219–1293 (75 bp).

### Phylogenetic Analysis of *penA* Alleles of Pen^NS^ Meningococci

To track the genetic origin of *penA* alleles of the Pen^NS^ isolates, we also analyzed the *penA* nucleotides of 724 commensal *Neisseria* isolates collected during 2013–2019. We found 288 *penA* alleles, all with the 5 common amino acid mutations in PBP2. The allele *penA*795 was the most frequent (8.6%, 62/724), followed by *penA964* (4.7%, 34/724) and *penA808* (4.4%, 32/724).

**Figure 3:**
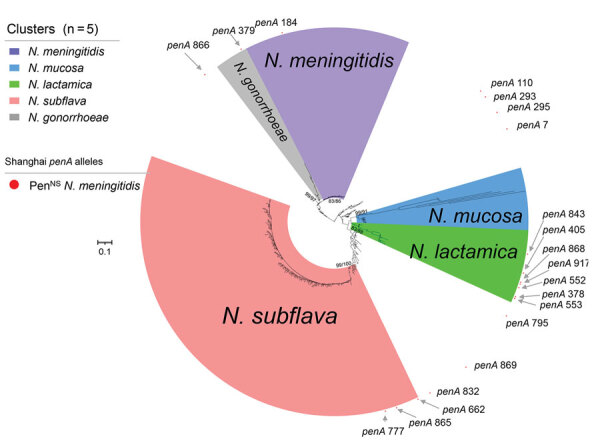
Phylogenetic analysis of *penA* alleles of *Neisseria* isolates and genomes, Shanghai, China, 1965–2020, and reference isolates. Phylogenetic analysis of the nucleotide sequences of 580 *penA* alleles (nucleotides 1321–1722) from *N. meningitidis* (n = 21,582), *N. gonorrhoeae* (n = 7,605), *N. lactamica* (n = 683), *N. subflava* (n = 431), *N. cinerea* (n = 65) , *N. polysaccharea* (n = 52), *N. mucosa* (n = 33), and other commensal *Neisseria* (n = 73) isolates and genomes collected in this study and from the *Neisseria* PubMLST database was constructed by using IQ-TREE version 2.2.0 (*23*), with both SH-aLRT test and UFboot set as 1,000. The values of SH-aLRT and ultrafast bootstrap (Ufboot) are shown on the node of each clade as SH-aLRT/Ufboot. Clusters were determined by using SH-aLRT values of 80% from the SH-aLRT tests with 1,000 replicates and ultrafast bootstrap (UFBoot) values of 85% from bootstrap tests with 1,000 replicates (IQ-TREE). Alleles *penA378*, *penA405*, *penA552*, *penA553*, *penA843*, *penA868*, and *penA917* were within in the *N. lactamica* cluster; *penA662*, *penA777*, and *penA865* were within the *N. subflava* cluster; *penA379* was within the *N. gonorrhoeae* cluster; and the other 8 *penA* alleles were located outside the 5 clusters. Scale bar indicates substitutions per site. Pen^NS^, penicillin-nonsusceptible meningococci.

For phylogenetic analysis, we used 580 *penA* alleles, including 250 *penA* alleles represented by 21,091 *N. meningitidis* and 8,218 commensal *Neisseria* genomes in the *Neisseria* PubMLST database, 20 alleles from Shanghai Pen^NS^ meningococci, 22 alleles from Shanghai penicillin-susceptible meningococci, and 288 alleles from Shanghai commensal *Neisseria* isolates. We found 5 clusters, corresponding to *N. meningitidis*, *N. lactamica*, *N. gonorrhoeae*, *N. mucosa*, and *N. subflava* (Figure 3).

Among the 20 *penA* alleles from the Shanghai Pen^NS^ meningococci, only *penA184* (A549T) was within the *N. meningitidis* cluster; the other 19 alleles (with >2 of the 5 mutations) scattered into the *N. lactamica* cluster (n = 7), the *N. subflava* cluster (n = 3), the *N. gonorrhoeae* cluster (n = 1), or outside the 5 clusters (n = 8). Those findings suggest that the PBP2 for the mutations was acquired by horizontal gene transfer (Figure 3).

### Crossover Point of Recombination Events in *penA*

Among the 19 Pen^NS^ meningococcal *penA* alleles acquired by horizontal gene transfer, we found that 6 *penA* alleles (*penA110*, *penA405*, *penA552*, *penA795*, *penA832*, and *penA843*) were shared by *N. meningitidis* and commensal *Neisseria* isolates (Appendix 2 Table 2). We analyzed 47 *Neisseria* genomes harboring these 6 alleles and found all potential donors of the 6 *penA* alleles; the sizes of the recombination fragments were 805–2,491 bp (Appendix 2 Table 2).

We discovered that the *penA795* allele, an allele associated with dual resistance to penicillin and 3GCs, was also harbored in the internationally disseminated ceftriaxone-resistant *N. gonorrhoeae* FC428 clone (Appendix 2 Table 2) (*25*). It was difficult to judge the origin donor of *penA795* because it was outside all the phylogenetic clusters and shared by 6 species of *Neisseria* (Appendix 2 Table 2).

### Genetic Transformation of *penA* Fragments with Mutations

Penicillin-susceptible *N. meningitidis* isolate Nm040 (B:P1.20,13-1:F5-2:ST-5798[CC4821]) was transformed with the chromosomal DNA of 9 commensal *Neisseria* isolates, each of which was considered to be 1 potential donor of the 5 meningococcal *penA* alleles (*penA405*, *penA552*, *penA795*, *penA832*, and *penA843*) (Appendix 2 Table 2). Transformants each acquired a *penA* allele the same as that of the corresponding donor isolate, leading to increased penicillin MICs from 0.032 μg/mL to 0.125-0.38 μg/mL. The lengths of the recombinant fragments carrying the partial or entire *penA* gene ranged from 512 to 10,534 bp (Appendix 2 Table 3). All transformants with *penA795* also acquired additional mutations (A311V, T483S, and N512Y), showing resistance to cefotaxime (0.25 or 0.5 μg/mL) (Appendix 2 Table 3).

The *penA* fragments (nucleotides 1237–1751) from 2 penicillin-intermediate meningococcal isolates (MIC 0.125 μg/mL) with only 1 or 2 mutations in PBP2, Nm469 (A549T) and Nm465 (F504L and A510V), were also used for transformation. Two groups of transformants each acquired the same PBP2 mutation(s) as their corresponding donor strain, and the penicillin MIC increased to 0.125 μg/mL, without increased cefotaxime MIC (Appendix 2 Table 3).

## Discussion

Pen^NS^ *N. meningitidis* strains (MICs 0.25–0.5 μg/mL) were recovered as early as 1985 in Spain (*26*). We discovered more Pen^NS^ isolates from carriers after 2007 and from patients after 2013 in Shanghai, although penicillin-intermediate meningococcus arose initially in 1967 (MIC 0.125 μg/mL; *penA379*; carrier; Appendix 1 Table 2). Our study presents the increasing trend of penicillin nonsusceptibility among *N. meningitidis* isolates in China during 1965–2020 (Figure 1), which is also supported by data from the China CDC and the Zhejiang CDC (*15*–*17*). This trend is similar to trends in other parts of the world, such as North America (≈30%), Europe (≈40%), and Australia (≈90%) (*27*–*29*). The nonsusceptibility was mostly associated with the 5 mutations of PBP2, which were found in widespread Pen^NS^ isolates globally (*14*). Of note, we found 3 isolates with resistance to both penicillin and cefotaxime, together with reduced susceptibility to ceftriaxone, which is rare in *N. meningitidis* worldwide (*30*).

Ceftriaxone is structurally similar to cefotaxime, sharing an exact R1 side chain and similar molecular mechanisms of action. We identified several mutations in the C-terminal or transpeptidase domain of the mosaic PBP2 that were associated with reduced cephalosporin susceptibility (e.g., I312M, V316T, F504L, N512Y, and G545S) (*31*); mutations A311V and T483S were associated with conferring ceftriaxone-resistance to *N. gonorrhoeae*. The cefotaxime-resistant meningococci from China (that contained mutations A311V and T483S in PBP2) differed from the cefotaxime-resistant isolate from the United Kingdom (with PBP2 mutations A501T and D511V) (*12*) but were consistent with the internationally disseminated ceftriaxone-resistant *N. gonorrhoeae* FC428 clone (*25*). Both *penA795*-bearing isolates Nm507 and FC428 had identical transpeptidase domains of PBP2 (Appendix 2 Figure 3), whereas FC428 displayed a 4-fold higher ceftriaxone MIC than Nm507, possibly resulting from the alterations in *penB* and the promoter region of *mtrR* in FC428 (*32*). We discovered that *penA795* was predominant in commensal *Neisseria* isolates in Shanghai, disseminated among *N. lactamica*, *N. cinerea*, *N. polysaccherae*, *N. subflava*, and *N. gonorrhoeae*. Phylogenetic analysis and genetic transformation suggest that the meningococcal cefotaxime resistance probably originates from *N. subflava* (*penA777* and *penA865*) and another unknown *Neisseria* (*penA795*).

As a hyperinvasive lineage first identified in the NmC meningococcal outbreaks in Anhui, China, during 2003–2005 (*3*), CC4821 has been challenging the preventive strategy of IMD in China for the past 2 decades. After the Anhui outbreaks, the national predominant serogroup shifted from NmA (>95%) before 2000 to NmC (43.3%) and NmA (36.8%) during 2005–2010; in response, in 2008, bivalent NmA and NmC meningococcal polysaccharide vaccine (MPV-AC) was introduced into the Expanded Programme for Immunization in China (*3*,*33*). In 2015, a total of 79% of CC4821 isolates were reported to possess quinolone resistance, and it was recommended that ciprofloxacin not be used as chemoprophylaxis for IMD in 2021 (*5*,*16*). In our study, we observed an increasing trend for acquisition of penicillin nonsusceptibility and cefotaxime resistance in quinolone-resistant CC4821 isolates, which further narrowed the choices for antimicrobial treatment and prophylaxis; safe and effective alternatives such as ceftriaxone, rifampin, and azithromycin could be considered to deal with this hyperinvasive lineage. Another concern is that NmB has become dominant in penicillin- and quinolone-resistant strains, accompanied by increasing nongroupable or rare serogroups (such as NmY and NmW), which could not be protected at present by vaccines in the Expanded Programme for Immunization in China (*33*).

Widespread resistance to either penicillin or ciprofloxacin, which is often associated with emergence of new resistant clones, has challenged the local strategies for treating and preventing IMD. After 2016, a new penicillin-resistant clade of W:P1.5,2:F1-1 (CC11) expanded from Australia to Europe and North America (*13*,*34*). In 2021, a *bla*_ROB-1_–containing Y:P1.5-2,10-2:F4-1:ST-3587(CC23) clone, which showed dual resistance to penicillin and ciprofloxacin, was identified in the United States and El Salvador (*35*,*36*). Among global CC4821 isolates, 2 antimicrobial-resistant clones were discovered: one is China^CC4821-R1-C/B^ (quinolone-resistant, *gyrA71*, NmC and NmB), expanding from China to other countries, and the other is the Europe-USA CC4821 cluster (Pen^NS^, *penA9*, NmB), which was restricted to countries outside of China (*7*). In our study, we observed rapid increases of the Pen^NS^ meningococcal strains with diversified Pen^NS^ alleles, which should be attributable to the selective pressure of penicillin after the increased consumption of broad-spectrum penicillin as indicated by the genetic diversity of these strains. Of note, 42.3% of the Pen^NS^ isolates and 2/3 of the cefotaxime-resistant isolates were assigned to the CC4821 cluster (Figure 2). Among the 11 Pen^NS^ isolates in the CC4821 cluster, 7 isolates were assigned to the same sublineage, L44.2. Among the L44.1 sublineage (identical to the hyperinvasive epidemic clone, China^CC4821-R1-C/B^), penicillin-, cefotaxime-, and quinolone-resistant ST-4821 strains have caused IMD, which should raise more concerns for public health.

Most (25/26) of the Pen^NS^ isolates in our study (Appendix 1 Table 2) are not covered by the scheduled meningococcal vaccines (MPV-A and MPV-AC) in China (*33*). All 3 cefotaxime-resistant isolates were from toddlers, who were unable to obtain protection from the corresponding vaccines (NmC or NmB) according to scheduled vaccination in China (*33*). MPV-AC is used only for children >3 years of age, and no NmB vaccines are available nationwide. To protect young children from cefotaxime-resistant isolates, on one hand, serogroup A and C meningococcal polysaccharide conjugate vaccine could be a good choice because it can cover populations >3 months of age (*37*); on the other hand, it is necessary to introduce or develop NmB vaccines for CC4821 strains from China.

In our study, 18/20 *penA* alleles identified in the Pen^NS^ isolates harbored the 5 penicillin-resistance-associated mutations in PBP2, and no prevalent alleles were found. In Europe and the United States, *penA12* (8%), *penA14* (6%), and *penA9* (5%) were the most prevalent alleles in Pen^NS^ isolates (*14*), but none of them were observed in isolates from China.

Phylogenetic analysis showed that most of the altered *penA* fragments of the Pen^NS^ isolates from China were acquired by horizontal gene transfer and most likely from *N. lactamica*, *N. subflava*, and *N. gonorrhoeae*. Analysis of >700 local commensal *Neisseria* isolates showed that their PBP2 all harbored the 5 common mutations, which could provide *N. meningitidis* isolates with various mutation-harboring *penA* alleles. On the basis of 6 *penA* alleles shared by *N. meningitidis* and commensal *Neisseria* isolates, those potential horizontal gene transfer events were validated by sequence analysis and genetic transformation (Appendix 2 Tables 2, 3).

One limitation of this study is the limited number of IMD isolates, which is mainly attributable to the recent low and decreasing incidence of IMD in China, from 0.18 cases (2005) to 0.0078 cases (2015–2019) per 100,000 population (*33*,*38*). Nevertheless, the isolates were phylogenetically related to the invasive meningococci in China, possessing representative features as demonstrated previously (*5*,*7*,*39*,*40*). The trend of increasing Pen^NS^ meningococci in China provided additional evidence for this study (*15*–*17*). Another limitation is that the *penA184* and *penA866* alleles were represented by only 1 penicillin-intermediate isolate each, which did not meet the requirements for the definition of Pen^NS^ *penA* alleles (*14*), although genetic transformations supported the contributions to the phenotype.

In summary, our study detected an ongoing shift in the penicillin susceptibility of meningococcal isolates in Shanghai. Pen^NS^ meningococcal isolates have increased in recent years, and Pen^NS^ CC4821 isolates have become predominant. Resistant *penA* alleles have been captured by quinolone-resistant CC4821 hyperinvasive epidemic clone with serogroup B or C. Because we do not yet have NmB vaccines with high coverage for NmB isolates of China^CC4821-R1-C/B^, the concern is that the triple-resistant CC4821 clone has the potential to cause an epidemic. The altered *penA* of Pen^NS^ isolates originated mainly from commensal *Neisseria* isolates, including *N. lactamica* and *N. subflava*. As part of the increasing trend of penicillin nonsusceptibility among *N. meningitidis* isolates in China during 1965–2020, quinolone-resistant CC4821 has acquired penicillin and cefotaxime resistance closely related to the internationally disseminated ceftriaxone-resistant gonococcal FC428 clone. 

Appendix 1 Additional information for study of penicillin and cefotaxime resistance of quinolone-resistant *Neisseria meningitidis* clonal complex 4821, Shanghai, China, 1965–2020.

Appendix 2 Supplemental results from study of penicillin and cefotaxime resistance of quinolone-resistant *Neisseria meningitidis* clonal complex 4821, Shanghai, China, 1965–2020.
