# Supplementary material for: Penicillin and Cefotaxime Resistance of Quinolone-Resistant Neisseria meningitidis Clonal Complex 4821, Shanghai, China, 1965–2020
Source: Emerg Infect Dis. 2023 Feb;29(2):341–50. doi: 10.3201/eid2902.221066 (PMC9881793; doi:10.3201/eid2902.221066)
Supplement: Appendix 2 — Supplemental results from study of penicillin and cefotaxime resistance of quinolone-resistant Neisseria meningitidis clonal complex 4821, Shanghai, China, 1965–2020. [file 22-1066-Techapp-s2.pdf]

Article DOI: https://doi.org/10.3201/eid2902.221066

Penicillin and Cefotaxime Resistance of Quinolone-Resistant *Neisseria meningitidis* Clonal Complex 4821, Shanghai, China, 1965–2020

Appendix

Appendix 2 Table 1. Characteristics of 3 cefotaxime-resistant meningococcal isolates, Shanghai, China*

Isolate	Year	Source	Age	Vaccination (MPV)	Sero-group	Sequence type	cgMLST cluster	PorA	FetA-VR	Ciprofloxacin MIC	Cefotaxime MIC, µg/mL	Ceftriaxone MIC, µg/mL	*penA* allele†	*penA* phylogenetic cluster
Nm462	2017	IMD	11 m	A (2 doses)	B	ST-7962	CC4821 cluster (L44.2)	P1.12-1,13-2	F4-21	0.5 (R)	0.5 (R)	0.064 (S)	777	*N. subflava*
Nm463	2017	carriage	3	A (2 doses), A+C (1 dose)	B	ST-13501	others	P1.21-2,28	F5-64	0.25 (R)	0.25 (R)	0.094 (S)	865	*N. subflava*
Nm507	2019	IMD	2	A (2 doses)	C	ST-4821	CC4821 cluster (L44.1)	P1.21-2,9	F3-3	0.5 (R)	0.25 (R)	0.125 (S)	795	none

* IMD, invasive meningococcal disease; MPV, meningococcal polysaccharide vaccine; ST, sequence type.

†These three alleles all possess 7 mutations in the penicillin binding-protein 2 (PBP2), including A311V, T483S, F504L, A510V, I515V, H541N, and I566V.

Appendix 2 Table 2. Crossover points of recombination events in *penA* alleles shared by *Neisseria meningitidis* and commensal *Neisseria* species

penA allele	Cluster	Donor species	Donor [isolate (PubMLST id, Country)]	Recombinant, isolate (PubMLS id)	Crossover point*		Fragment length (bp)†
					Upstream	Downstream	
*penA*110	None	*N. lactamica*	OX40308 (44473, UK)	Nm054 (19516)	718	2048	1331
		N. cinerea	LNP28127 (42057, France)	Nm054 (19516)	718	2357	1640
		N. polysaccharea	M15 240880 (41474, UK)	Nm054 (19516)	742	2150	1409
		N. bergeri	01_00007_XS1_1 (43428, Mali)	Nm054 (19516)	718	2153	1436
*penA*405	*N. lactamica*	*N. lactamica*	Nei171 (105709, China)	Nm075 (52223)	1000	1804	805
		N. lactamica	Nei157 (108165, China)	Nm395 (52373), Nm479 (72258)	873	3199	2327
*penA*552	*N. lactamica*	*N. lactamica*	Nei033 (84202, China)	Nm393 (52746)	913	1976	1064
		N. lactamica	Nei033 (84202, China)	Nm464 (58441)	859	1804	946
*penA*795	None	*N. lactamica*	Nei012 (84195, China)	Nm507 (72262)	691	1819	1132
		N. cinerea	Nei028 (84201, China)	Nm507 (72262)	892	1819	931
		N. polysaccherae	Nei367 (84258, China)	Nm507 (72262)	532	1819	1291
		N. subflava	Nei798 (105711, China)	Nm507 (72262)	532	1819	1291
		N. gonorrhoeae	FC428 (108261, Japan)	Nm507 (72262)	907	1819	916
*penA*832	None	*N. lactamica*	Nei386 (108166, China)	Nm424 (52183)	709	3199	2491
		N. lactamica	Nei341 (83066, China)	Nm512 (71401)	718	2094	1377

penA allele	Cluster	Donor species	Donor [isolate (PubMLST id, Country)]	Recombinant, isolate (PubMLS id)	Crossover point* Upstream	Crossover point* Downstream	Fragment length (bp)†
*penA*843	*N. lactamica*	*N. lactamica*	Nei264 (105710, China)	Nm419 (55186), Nm514 (106175)	233	1908	1679
		N. cinerea	Nei1004 (105712, China)	Nm419 (55186), Nm514 (106175)	922	1793	875

*Positions are given according to the first nucleotide of *penA* of *N. meningitidis* strain 053442.

†The length is determined according to the donor strain.

Appendix 2 Table 3. Characteristics of isolates in the genetic transformation into *Neisseria meningitidis* isolates*

Attribute of strain	Strain	Species	MIC, μg/mL Penicillin	MIC, μg/mL Cefotaxime	*penA* allele (mutation)†	Crossover point‡ Upstream	Crossover point‡ Downstream	Length of recombinant fragment (bp)§	PubMLST ID
Recipient	Nm040	*N. meningitidis*	0.032	0.008	1 (none)	NA	NA	NA	58130
Donor 1	Nei171	*N. lactamica*	0.125	0.06	405 (5 mutations)	NA	NA	NA	105709
Transformant 1.1	Nm040Nei171T1-1	*N. meningitidis*	0.125	0.06	405 (5 mutations)	742	3098	2357	111282
Transformant 1.2	Nm040Nei171T1-2	*N. meningitidis*	0.19	0.03	405 (5 mutations)	242	2697	2456	111283
Transformant 1.3	Nm040Nei171T1-3	*N. meningitidis*	0.19	0.06	405 (5 mutations)	343	2953	2611	111284
Donor 2	Nei033	*N. lactamica*	0.5	0.06	552 (5 mutations)	NA	NA	NA	84202
Transformant 2.1	Nm040Nei033T1-1	*N. meningitidis*	0.38	0.015	552 (5 mutations)	913	7393	5740	111279
Transformant 2.2	Nm040Nei033T1-2	*N. meningitidis*	0.125	0.015	552 (5 mutations)	913	5330	3765	111280
Transformant 2.3	Nm040Nei033T1-3	*N. meningitidis*	0.19	0.015	552 (5 mutations)	913	6877	5303	111281
Donor 3	Nei012	*N. lactamica*	1.5	1	795 (7 mutations)	NA	NA	NA	84195
Transformant 3.1	Nm040Nei012T1-6	*N. meningitidis*	0.25	0.5	795 (7 mutations)	718	2999	2285	111273
Transformant 3.2	Nm040Nei012T1n-2	*N. meningitidis*	0.19	0.5	795 (7 mutations)	817	9473	8628	111274
Transformant 3.3	Nm040Nei012T1n-3	*N. meningitidis*	0.25	0.5	795 (7 mutations)	262	7393	6443	111275
Donor 4	Nei028	*N. cinerea*	1	1	795 (7 mutations)	NA	NA	NA	84201

Attribute of strain	Strain	Species	MIC, µg/mL Penicillin	Cefo-taxime	*penA* allele (mutation)†	Crossover point‡ Upstream	Down-stream	Length of recombinant fragment (bp)§	PubMLST ID
Transformant 4.1	Nm040Nei028T1n-1	*N. meningitidis*	0.38	0.5	795 (7 mutations)	-1489	6412	7215	111276
Transformant 4.2	Nm040Nei028T1n-2	*N. meningitidis*	0.25	0.5	795 (7 mutations)	643	1976	1337	111277
Transformant 4.3	Nm040Nei028T1n-3	*N. meningitidis*	0.25	0.5	795 (7 mutations)	864	2999	2139	111278
Donor 5	Nei367	*N. polysaccherae*	0.75	0.5	795 (7 mutations)	NA	NA	NA	84258
Transformant 5.1	Nm040Nei367T1-1	*N. meningitidis*	0.25	0.25	795 (7 mutations)	-1242	10501	10534	111288
Transformant 5.2	Nm040Nei367T1-2	*N. meningitidis*	0.38	0.25	795 (7 mutations)	85	596	512	111289
Transformant 5.3	Nm040Nei367T1-3	*N. meningitidis*	0.19	0.25	795 (7 mutations)	-285	3011	3299	111290
Donor 6	Nei798	*N. subflava*	0.25	0.25	795 (7 mutations)	NA	NA	NA	105711
Transformant 6.1	Nm040Nei798T1-1	*N. meningitidis*	0.19	0.25	795 (7 mutations)	532	2774	2246	111291
Transformant 6.2	Nm040Nei798T1-3	*N. meningitidis*	0.25	0.25	795 (7 mutations)	873	6294	3698	111292
Transformant 6.3	Nm040Nei798T1-4	*N. meningitidis*	0.25	0.25	795 (7 mutations)	418	2459	2045	111293
Donor 7	Nei341	*N. lactamica*	0.5	0.25	832 (5 mutations)	NA	NA	NA	83066
Transformant 7.1	Nm040Nei341T1-1	*N. meningitidis*	0.19	0.125	832 (5 mutations)	757	2048	1292	111265
Transformant 7.2	Nm040Nei341T1-2	*N. meningitidis*	0.19	0.125	832 (5 mutations)	328	8664	7805	111266
Transformant 7.3	Nm040Nei341T1-3	*N. meningitidis*	0.19	0.125	832 (5 mutations)	328	5534	4554	111267
Donor 8	Nei264	*N. lactamica*	0.75	0.015	843 (5 mutations)	NA	NA	NA	105710
Transformant 8.1	Nm040Nei264T1-1	*N. meningitidis*	0.25	0.015	843 (5 mutations)	475	3161	2690	111285
Transformant 8.2	Nm040Nei264T1-2	*N. meningitidis*	0.19	0.015	843 (5 mutations)	348	2693	2349	111286
Transformant 8.3	Nm040Nei264T1-3	*N. meningitidis*	0.125	0.015	843 (5 mutations)	348	2693	2349	111287
Donor 9	Nei1004	*N. cinerea*	0.5	0.015	843 (5 mutations)	NA	NA	NA	105712
Transformant 9.1	Nm040Nei1004T1-1	*N. meningitidis*	0.25	0.015	843 (5 mutations)	532	1976	1448	111294
Transformant 9.2	Nm040Nei1004T1-2	*N. meningitidis*	0.25	0.015	843 (5 mutations)	532	1976	1448	111295
Transformant 9.3	Nm040Nei1004T1-3	*N. meningitidis*	0.125	0.015	843 (5 mutations)	895	3199	2308	111296
Donor 10	Nm469	*N. meningitidis*	0.125	0.008	184 (A549T)	NA	NA	NA	105710
Transformant 10.1	Nm040Nm469T1-1	*N. meningitidis*	0.125	0.008	184 (A549T)	1645	1645	1	116982

Attribute of strain	Strain	Species	MIC, μg/mL Penicillin	Cefo-taxime	*penA* allele (mutation)†	Crossover point‡ Upstream	Down-stream	Length of recombinant fragment (bp)§	PubMLST ID
Transformant 10.2	Nm040Nm469T1-3	*N. meningitidis*	0.125	0.008	184 (A549T)	1645	1645	1	116983
Transformant 10.3	Nm040Nm469T1-4	*N. meningitidis*	0.125	0.008	184 (A549T)	1645	1645	1	116984
Donor 11	Nm465	*N. meningitidis*	0.125	0.008	866 (F504L and A510V)	NA	NA	NA	105712
Transformant 11.1	Nm040Nm465T1-1	*N. meningitidis*	0.125	0.008	new1 (F504L and A510V) ¶	1467	1533	67	116985
Transformant 11.2	Nm040Nm465T1-5	*N. meningitidis*	0.125	0.008	new2 (F504L and A510V)#	1431	1533	103	116986

*NA, not applicable.

†The 5 mutations are F504L, A510V, I515V, H541N, and I566V; and the 7 mutations are A311V, T483S, F504L, A510V, I515V, H541N, and I566V.

‡Positions are given according to the first nucleotide of *penA* gene of the *N. meningitidis* strain Nm040. NA, not applicable.

§The length is determined according to the donor strain.

¶The allele new1 and *penA866* differ in 2 nucleotides.

#The allele new2 and *penA866* differ in 1 nucleotide.

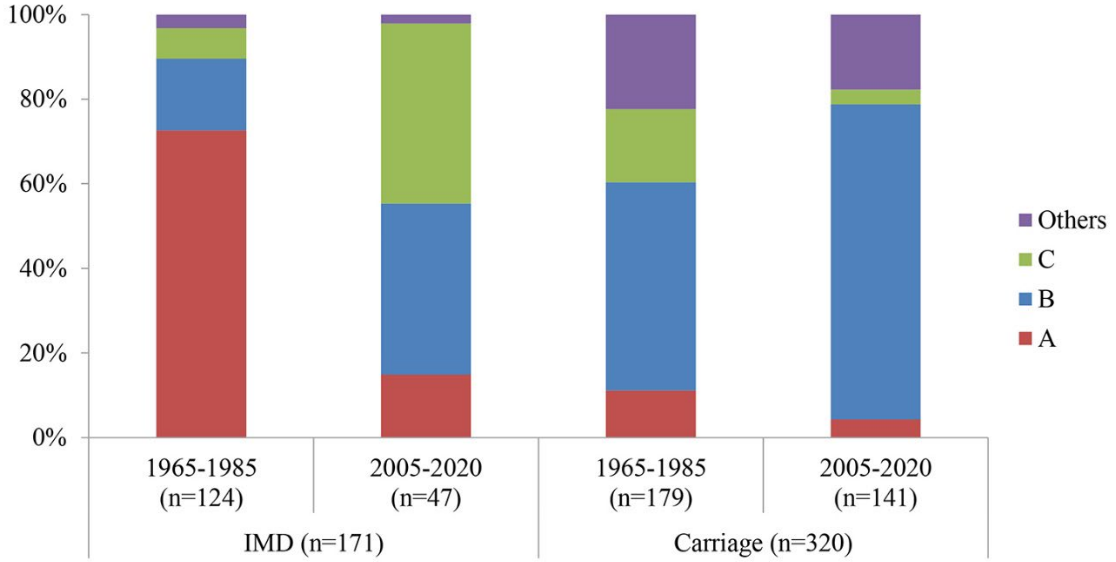

Appendix 2 Figure 1. Serogroup distribution of invasive and carriage meningococcal isolates in Shanghai during 1965-1985 and 2005-2020. IMD, invasive meningococcal disease. A total of 491 *N. meningitidis* isolates were collected from 1965 to 2020, including 234 NmB (47.7%), 123 NmA (25.1%), 65 NmC (13.2%), 31 nongroupable (6.3%), 13 NmE (2.6%), 11 NmY (2.2%), 7 NmW (1.4%), 4 NmZ (0.8%), and 3 NmX (0.6%).

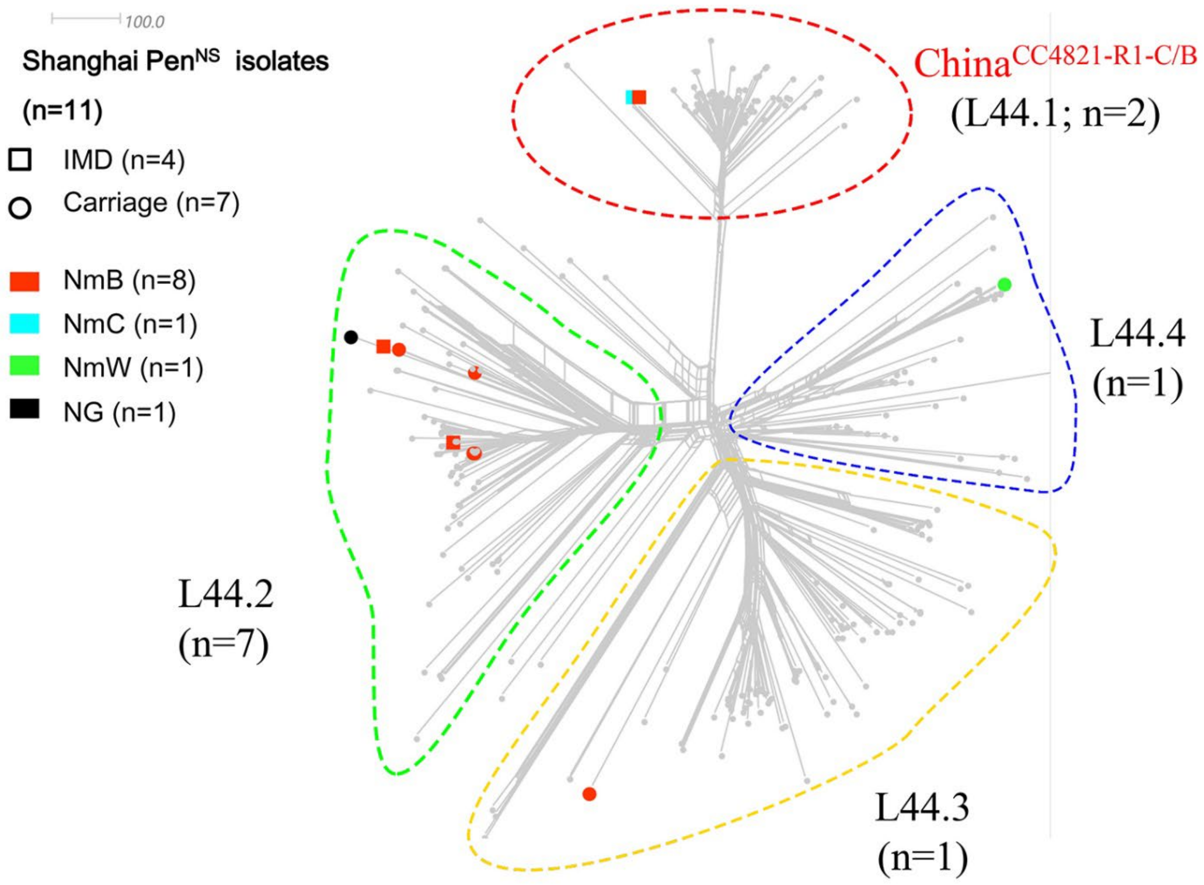

Appendix 2 Figure 2. Distribution of CC4821 penicillin non-susceptible isolates in the four sublineages of CC4821 by genome analysis. Pen^{NS}, penicillin non-susceptible. IMD, invasive meningococcal disease. NmB, serogroup B. NmC, serogroup C. NmW, serogroup W. NG, nongroupable.

A

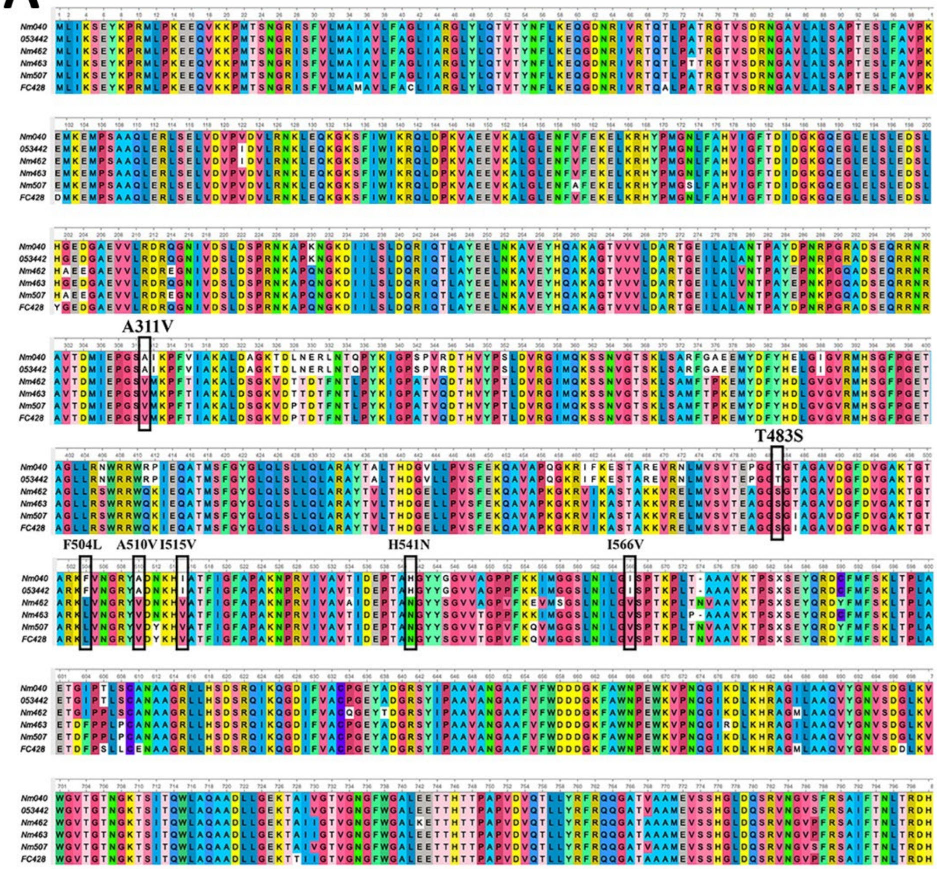
Nm040
053442
Nm462
Nm463
Nm507
FC428
A311V
T483S
F504L
A510V
I515V
H541N
I566V

B

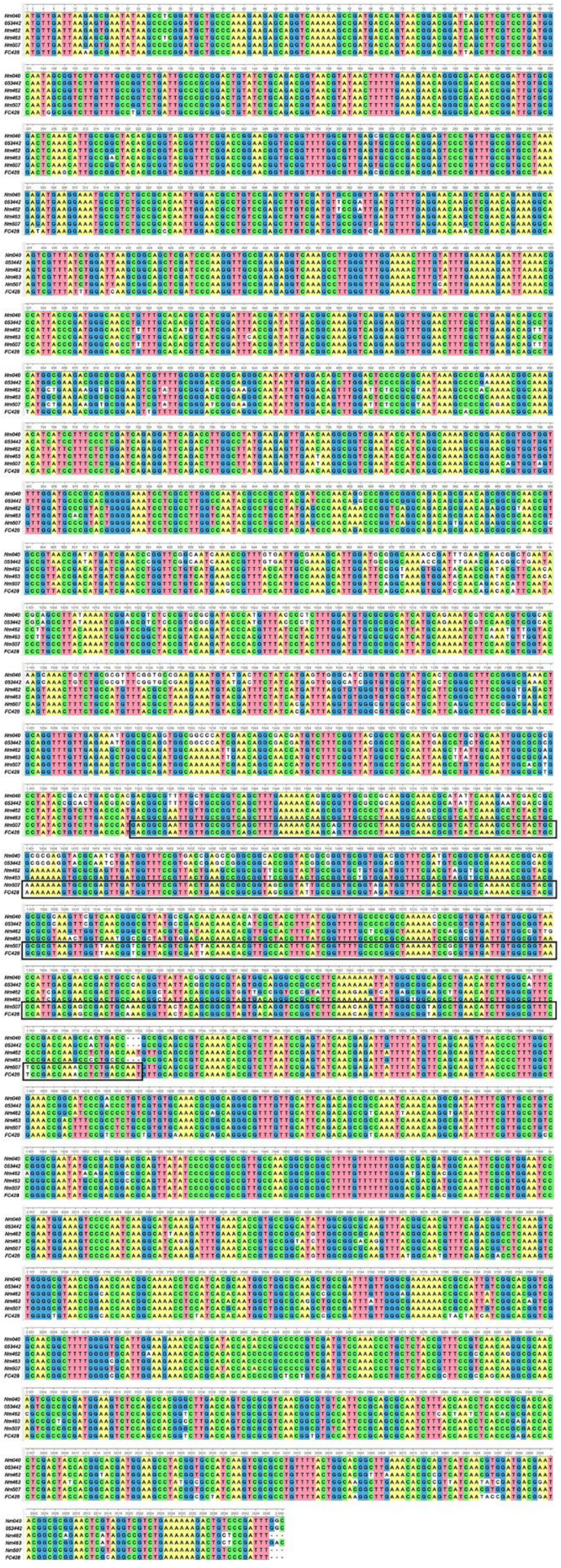

Appendix 2 Figure 3. Alignment of the *penA* full-length sequences of the three cefotaxime-resistant isolates, two wild-type *N. meningitidis* strains, and the *N. gonorrhoeae* FC428 isolate. A) Amino acid sequences. The two mutations associated with cefotaxime-resistance (A311V and T483S) and the five mutations associated with penicillin-resistance (F504L, A510V, I515V, H541N, and I566V) are indicated. B) Nucleotides. The 402bp-length sequences of isolates Nm507 and FC428 used to define *penA* allele are indicated by black box.
